# Health care access barriers among metropolitan and nonmetropolitan populations of eight geographically diverse states, 2018

**DOI:** 10.1111/jrh.12855

**Published:** 2024-06-14

**Authors:** Whitney E. Zahnd, Peiyin Hung, Elizabeth L. Crouch, Radhika Ranganathan, Jan M. Eberth

**Affiliations:** ^1^ Department of Health Management and Policy, College of Public Health University of Iowa Iowa City Iowa USA; ^2^ Rural and Minority Health Research Center, Arnold School of Public Health University of South Carolina Columbia South Carolina USA; ^3^ Department of Health Management and Policy, Dornsife School of Public Health Drexel University Philadelphia Pennsylvania USA

**Keywords:** access to care, health disparities, rural health

## Abstract

**Introduction:**

Nonmetropolitan populations face frequent health care access barriers compared to their metropolitan counterparts, but differences in the number of these barriers across groups are not known. Our objective was to examine the differences in health care access barriers across metropolitan, micropolitan, and noncore populations.

**Methods:**

We used Behavioral Risk Factor Surveillance System data from the optional “Health Care Access” module to perform a cross‐sectional analysis examining access barriers across levels of rurality using bivariate analyses and Poisson models. Access barriers were operationalized as a count ranging from 0 to 5, reflective of the number of financial barriers and nonfinancial barriers.

**Results:**

Micropolitan and noncore respondents had lower educational attainment, were older, and were less racially/ethnically diverse than metropolitan respondents. They also reported more barriers, including lacking health insurance, medical debt, and foregoing care or medication due to cost. These barriers were most pronounced in non‐Hispanic Black, Hispanic, and American Indian/Alaska Native nonmetropolitan populations, compared to their White counterparts. In adjusted analysis, micropolitan respondents reported more barriers compared to metropolitan (prevalence rate ratio = 1.06; 95% confidence interval: 1.02–1.10) as did women, racial/ethnic minority populations, and those with less education.

**Conclusions:**

Micropolitan populations experience more barriers to health care, and nonmetropolitan respondents report more cost‐related barriers than their metropolitan counterparts, raising concerns on health care disparities and financial burdens for these underserved populations. This underscores the need to mitigate these barriers, particularly among those in micropolitan areas and minorized populations.

## INTRODUCTION

Both access to and quality of health care services play important roles in mental and physical well‐being and health outcomes.^1^ Geographic maldistribution to health care access contributes to disparities in health outcomes between nonmetropolitan and metropolitan residents.^2^ There are more than 61 million nonmetropolitan residents,^3^ yet health care services are less available and accessible to nonmetropolitan residents due to higher provider‐to‐population ratios and the need to travel greater distances to receive care.^2^, ^3^, ^4^, ^5^, ^6^, ^7^, ^8^ Additionally, nonmetropolitan hospitals are closing at an alarming rate, further reducing the availability of emergency care and other health care services.^9^, ^10^

Disparities in nonmetropolitan/metropolitan access to health care services are further exacerbated among racial/ethnicity minority individuals.^10^, ^11^, ^12^, ^13^, ^14^ Compared to their White counterparts, racial/ethnic minority residents experience racism and greater exposure to structural disadvantage leading to poorer health outcomes than White residents, with pronounced differences in morbidity and mortality.^11^, ^12^, ^13^, ^14^ Prior research demonstrates that nonmetropolitan racial/ethnic minority populations, such as Black or American Indian/Alaska Native residents, are much less likely to have equitable access to care than their White counterparts.^10^

Penchansky and Thomas developed a now commonly used operationalization of access to health care as the “5 A's of access”, including availability, accessibility, affordability, accommodation, and acceptability.^15^, ^16^ Availability considers whether a certain type of facility/provider is within a geographic area of interest (e.g., a hospital in a county or a physician‐to‐population ratio). Accessibility considers the distance or travel time to a facility or provider (e.g., miles to the nearest hospital). Affordability addresses cost of care (e.g., whether someone has insurance coverage, out of pocket expenses). Accommodation refers to whether the provider organization is accommodating to patient needs and education (e.g., how appointment scheduling occurs, availability of transportation services, offers language translation). Acceptability refers to whether the services provided were done so to the satisfaction of the patient.^17^

These measures of access are important to develop and adapt policies and programs among populations who are medically underserved. Studies of access to care among nonmetropolitan and racial/ethnic minorities have frequently examined availability, accessibility, and affordability.^2^, ^3^, ^4^, ^5^, ^6^, ^7^, ^8^, ^18^ Individual studies on nonmetropolitan/metropolitan differences in accommodation and acceptability have thus far had very mixed findings, and studies on acceptability have often been limited to Medicare beneficiaries or other older adult groups, limiting their generalizability.^19^, ^20^, ^21^, ^22^ However, there is limited research on how the number of access to care barriers differs across metropolitan and nonmetropolitan populations and among nonmetropolitan, minorized populations. Understanding barriers across multiple domains in diverse populations and geographies can help identify populations with the greatest needs. Therefore, the purpose of this study was to examine differences in barriers to access considering the number of both financial (affordability) and nonfinancial (accommodation) barriers to care across noncore, micropolitan, and metropolitan populations using measures available in the Behavioral Risk Factors Surveillance System (BRFSS). The findings from this study will be instrumental for policymakers and program planners as they develop and implement strategies to improve health outcomes among nonmetropolitan residents and reduce geographic disparities.

## METHODS

Publicly available data from the 2018 BRFSS were used for this study. BRFSS is an annual survey (landline and cellphone) that collects information on health‐related risk behaviors, chronic health conditions, and the utilization of preventive services. The survey is administered by each state in partnership with the Centers for Disease Control and Prevention's (CDC's) Division of Behavioral Surveillance, Office of Surveillance, Epidemiology and Laboratory Services. Respondents must be noninstitutionalized US adults who are aged 18 or older at the time of the interview. There are a core set of questions with individual states having the option to include additional modules on topics of interest. The health care access module, utilized in this study, included eight states (Georgia, Louisiana, Mississippi, Nebraska, New Hampshire, New Mexico, Oregon, and Tennessee) that opted to offer this module in 2018. We use 2018 data only as they represent the most expansive set of questions in the optional module that both includes a comprehensive geographic indicator and reflects the respondent experiences unimpacted by the COVID‐19 pandemic. In years prior to 2018, the geographic indicator was available only for those completing the survey via a landline, which is a much smaller sample. Additionally, while the health access module was available in the prepandemic year of 2019, different and fewer questions were used.

From the health care access module questions, we summed the number of barriers indicated by survey participants considering the survey questions outlined in Table 1 aligned with either the affordability or accommodation components of the “5 A's of Access.” Potential count values ranged from 0 to 5, with higher counts signifying more access barriers.

**TABLE 1:** Participant characteristics.

	Noncore	Micropolitan	Metropolitan	*p*‐value
	*N* (weighted %)			
Total (row %)	9,433 (10.0%)	13,803 (16.1%)	31,261 (73.9%)	N/A
Demographic characteristics				
Race/ethnicity (imputed)				<0.001
Non‐Hispanic White	7,746 (72.4%)	10,377 (71.5%)	22,598 (64.3%)	
Non‐Hispanic Black	959 (19.3%)	1,487 (15.2%)	3,911 (21.0%)	
Asian/Pacific Islander	21 (0.5%)	53 (0.6%)	442 (2.4%)	
American Indian/Alaska Native	173 (1.2%)	399 (2.2%)	575 (1.2%)	
Hispanic	397 (5.2%)	1,226 (8.8%)	2,9,30 (8.8%)	
Non‐Hispanic/other	137 (1.5%)	261 (1.8%)	805 (2.3%)	
Age				<0.001
18–64	5,710 (74.0%)	8,632 (76.0%)	21,250 (79.4%)	
65 and older	3,723 (26.0%)	5,171 (24.0%)	10,011 (20.6%)	
Gender				0.99
Male	4,037 (47.6%)	5,953 (47.8%)	13,940 (47.4%)	
Female	5387 (52.3%)	7,824 (52.0%)	17,264 (52.4%)	
Don't know/refused/missing	9 (0.2%)	26 (0.2%)	57 (0.2%)	
Marital status				<0.001
Married	5,278 (51.8%)	7,168 (50.6%)	15,956 (50.3%)	
Divorced/Separated	1,202 (12.4%)	2,072 (13.1%)	4,541 (11.5%)	
Widowed	1,416 (10.0%)	1,775 (8.4%)	3244 (6.7%)	
Never married	1,293 (22.1%)	2,403 (25.2%)	6,502 (28.1%)	
Don't know/missing	28 (0.5%)	62 (0.4%)	216 (0.6%)	
Education level				<0.001
Less than high school	916 (19.3%)	1,308 (16.0%)	2,522 (12.3%)	
High school graduate	3,281 (36.2%)	4,280 (33.0%)	8,166 (28.2%)	
Attended college/technical school	2,797 (28.8%)	3,942 (31.9%)	8,487 (31.6%)	
College/technical school graduate	2,414 (15.4%)	4,239 (18.9%)	11,986 (27.5%)	
Don't know/refused/missing	25 (0.3%)	–	100 (0.4%)	
Employment status				
Employed for wages	3,425 (38.7%)	5,267 (42.9%)	13,457 (47.0%)	<0.001
Self‐employed	1,267 (9.5%)	1,290 (9.0%)	2,786 (9.4%)	
Unemployed	255 (4.6%)	490 (5.0%)	1,254 (5.0%)	
Homemaker	510 (6.2%)	725 (5.9%)	1,500 (5.1%)	
Student	138 (3.1%)	258 (4.1%)	883 (4.6%)	
Retired	2,877 (22.3%)	4,267 (19.5%)	8,494 (14.0%)	
Unable to work	872 (14.3%)	1,391 (11.5%)	2,486 (8.8%)	
Don't know/refused/missing	89 (1.3%)	115 (1.0%)	401 (1.7%)	
Medicaid expansion status, yes	1,077 (18.0%)	6,825 (39.0%)	15,319 (37.7%)	<0.001
Individual barriers				
Have no current health insurance, yes	779 (13.1%)	1,291 (14.1%)	2,960 (12.4%)	0.01
Delayed care due to cost, yes	1057 (16.4%)	1,651 (15.8%)	3,791(14.9%)	0.09
Changes in medication adherence due to cost, yes	732 (10.9%)	1,155 (9.9%)	2,672 (9.2%)	0.02
Have medical bills you are paying off over time, yes	2,020 (25.1%)	2,956 (24.9%)	6,234 (22.7%)	<0.001
Experienced a non‐financial delay in care, yes	1,262 (17.8%)	2,314 (19.9%)	5,362 (19.4%)	0.11
At least one barrier	3,605 (47.1%)	5,795 (50.0%)	12,883 (46.1%)	<0.001

The National Center for Health Statistics’ Urban‐Rural Classification for Counties was used to categorize respondents as metropolitan, micropolitan, or noncore. *Race/ethnicity* was self‐reported by the survey respondents and classified as non‐Hispanic White, non‐Hispanic Black, Hispanic, American Indian/Alaska Native, Asian/ Pacific Islander, and other, which includes multiracial and nonspecified race. Other sociodemographic measures included in this study were sex, age, marital status, education level, and employment status. Imputed versions of race/ethnicity and age available in the BRFSS dataset were used to reduce missingness. We also categorized respondents by whether they lived in a state that had expanded Medicaid prior to 2018 when this iteration of BRFSS had been administered. Among those states that participated in the BRFSS health care access optional module, Louisiana, New Hampshire, New Mexico, and Oregon had expanded Medicaid, while Georgia, Mississippi, Nebraska, and Tennessee had not.

### Analysis

We calculated frequencies and weighted percentages across the three geographic groups for sociodemographic characteristics and experiences of individual barriers and compared them using Rao–Scott Chi‐square tests. We summed the number of barriers across metropolitan statuses and racial/ethnic groupings. Poisson regression models with robust standard errors were used to assess differences in the number of barriers across noncore, micropolitan, and metropolitan populations. We reported unadjusted and adjusted prevalence rate ratios (PRRs) and corresponding 95% confidence intervals (CIs). In our adjusted models, we control for sociodemographic variables: race/ethnicity, sex, age, marital status, education level, employment status, and Medicaid expansion status of respondent's state of residence. We accounted for the complex survey design in our analyses. All analyses included BRFSS calibration weights, including primary sampling units (*_PSU*), stage‐level sample weight (_*LLCPWT*), and sampling strata (_*STSTR*), and were performed using survey procedures. Analyses were performed in Stata.

## RESULTS

In the study sample, 10.0% of respondents were from noncore, 16.1% from micropolitan, and 73.9% from metropolitan areas (Table 2). More than 15% of the study sample was non‐Hispanic Black across all three geographic groups, though metropolitan respondents tended to be more racially/ethnically diverse. Noncore and micropolitan respondents tended to be older with 26.0% and 24.0% of respondents 65 years of age or older compared to 20.6% of metropolitan respondents (*p* < 0.001). Lower proportions of noncore (15.4%) and micropolitan (18.9%) were college or technical school graduates compared to metropolitan (27.5%; *p* < 0.001). Additionally, employment status varied by rurality, with 22.3% of noncore and 19.5% of micropolitan respondents being retired compared to 14.0% of metropolitan respondents (*p* < 0.001). More than a third of micropolitan (39.0%) and metropolitan (37.7%) respondents lived in states that had yet to expand Medicaid (*p* < 0.001).

**TABLE 2:** Associations between participant characteristics and access to health care barriers.

	Unadjusted prevalence rate ratios (95% CI)	Adjusted prevalence rate ratios (95% CI)	*p*‐value
Metropolitan status			
Nonmetro			
Nonmetro micropolitan	1.08 (1.03–1.13)	1.06 (1.02–1.10)	0.006
Nonmetro noncore	1.06 (1.00–1.13)	0.99 (0.94–1.05)	0.791
Metro	Reference	Reference	
Race/ethnicity			
Non‐Hispanic White	Reference	Reference	
Non‐Hispanic Black	1.38 (1.32–1.45)	1.12 (1.06–1.18)	<0.001
Asian/Pacific Islander	0.91 (0.73–1.12)	0.96 (0.78–1.19)	0.727
American Indian/Alaska Native	1.45 (1.29–1.64)	1.20 (1.06–1.35)	0.003
Hispanic	1.61 (1.53–1.69)	1.31 (1.24–1.38)	<0.001
Non‐Hispanic/other	1.43 (1.30–1.57)	1.32 (1.20–1.44)	<0.001
Age			
18–64	Reference	Reference	
65 and older	0.42 (0.40–0.45)	0.54 (0.50–0.57)	<0.001
Gender			
Male	Reference	Reference	
Female	1.21 (1.17–1.26)	1.23 (1.18–1.28)	<0.001
Don't know/refused/missing	1.04 (0.68–1.57)	1.15 (0.76–1.75)	0.508
Marital status			
Married	Reference	Reference	
Divorced/separated	1.55 (1.48–1.63)	1.31 (1.25–1.38)	<0.001
Widowed	0.92 (0.84–0.99)	1.12 (1.04–1.22)	0.005
Never married	1.48 (1.41–1.55)	1.19 (1.14–1.25)	<0.001
Don't know/missing	1.25 (0.98–1.60)	1.16 (0.93–1.47)	0.193
Education level			
Less than high school	2.21 (2.08–2.34)	1.80 (1.69–1.92)	<0.001
High school graduate	1.62 (1.54–1.70)	1.43 (1.36–1.50)	<0.001
Some college/technical school	1.52 (1.45–1.60)	1.41 (1.34–1.48)	<0.001
College/technical school graduate	Reference	Reference	
Don't know/refused/missing	1.42 (1.05–1.93)	1.27 (0.95–1.71)	0.106
Employment status			
Employed for wages	Reference	Reference	
Self‐employed	1.07 (1.01–1.15)	1.13 (1.06–1.20)	<0.001
Unemployed	1.88 (1.75–2.01)	1.60 (1.49–1.71)	<0.001
Homemaker	1.21 (1.12–1.31)	1.11 (1.03–1.21)	0.009
Student	0.95 (0.85–1.06)	0.83 (0.75–0.93)	0.001
Retired	0.48 (0.46–0.51)	0.75 (0.70–0.81)	<0.001
Unable to work	1.65 (1.56–1.74)	1.40 (1.32–1.48)	<0.001
Don't know/refused/missing	1.16 (1.00–1.35)	1.09 (0.94–1.25)	0.242
Medicaid expansion status			
Medicaid expansion states	Reference	Reference	
Non‐Medicaid expansion states	1.21 (1.17–1.26)	1.22 (1.17–1.27)	<0.001

*Notes* Both unadjusted and adjusted tables were estimated in weighted data using the Behavioral Risk Factor Surveillance System calibration weights, including primary sampling units (*_PSU*), stage‐level sample weight (_*LLCPWT*), and sampling strata (_*STSTR*).

Abbreviation: CI, confidence interval.

Higher percentages of noncore (13.1%) and micropolitan respondents (14.1%) did not currently have health insurance compared to metropolitan respondents (12.4%; *p* = 0.01; Table 1). Similarly, higher percentages of noncore (10.9%) and micropolitan respondents (9.9%) changed their adherence to prescribed and/or over‐the‐counter medications due to cost compared to metropolitan peers (9.2%; *p* = 0.02). Roughly a quarter of noncore (25.1%) and micropolitan (24.9%) respondents reported having medical bills they were paying off over time compared to 22.7% of metropolitan respondents (*p* < 0.001). There were no statistically significant differences in delaying care due to cost and experiencing a delay in care for a nonfinancial reason by rurality.

**FIGURE 1:**
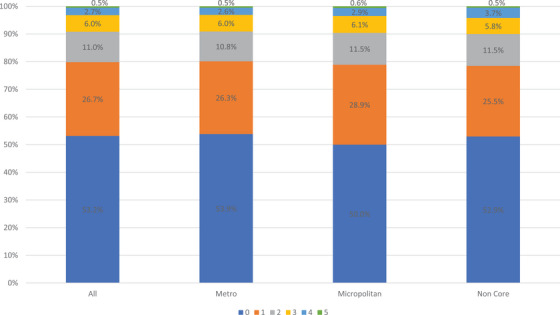
Number of health care access barriers among all respondents by metropolitan status.

In examining the number of barriers respondents experienced, we found that 53.3% of metropolitan respondents experienced no barriers compared to 50.0% of micropolitan respondents and 52.9% of noncore respondents (Figure 1). Furthermore, 37.1% of metropolitan respondents reported one to two barriers compared to 40.4% of micropolitan and 37.0% of noncore respondents. The majority of non‐White respondents reported at least one barrier across metropolitan, micropolitan, and noncore geographies (Figure 2). Among metropolitan respondents, a greater percent of Hispanics (13.9%) reported at least three barriers compared to other racial/ethnic groups (11.8% or lower; Figure 2A). Among micropolitan respondents, both non‐Hispanic Black and Black respondents (14.7% for both) had the highest rates of at least three barriers. Among noncore respondents, Hispanics reported the highest rates of at least three barriers (15.1%, Figure 2C).

FIGURE 2Number of health care access barriers by racial/ethnic groups: (A) metropolitan; (B) micropolitan; and (C) noncore.

**Figure:**
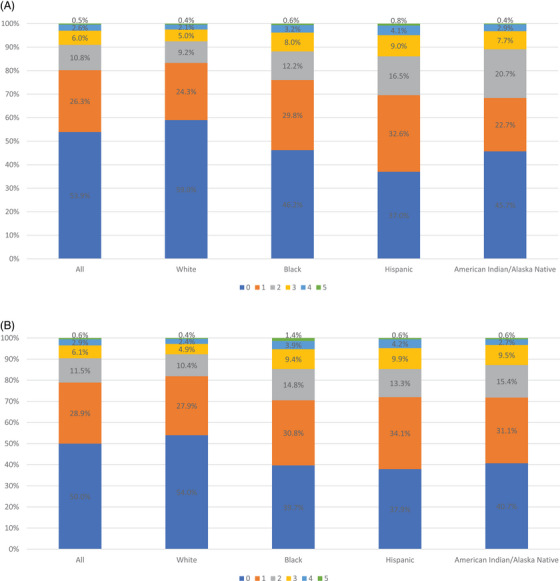

**Figure:**
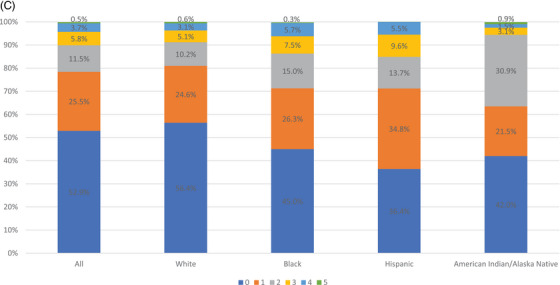

Unadjusted analysis showed higher PRRs in noncore (PRR = 1.06; 95% CI: 1.00–1.13) and micropolitan (PRR = 1.08; 95% CI: 1.03–1.13) respondents compared to metropolitan (Table 2). Unadjusted analysis also showed that, compared to non‐Hispanic White respondents, those of all other racial/ethnic groups except for Asian/Pacific Islanders experienced higher prevalence ratios. Additional differences were identified in unadjusted analysis by age, gender, marital status, age, employment status, educational status, and Medicaid expansion status.

In adjusted analysis, micropolitan respondents also experienced higher PRR (1.06; 95% CI: 1.02–1.10) compared to metropolitan respondents, but this association was attenuated among noncore respondents (Table 2). Adjusted analysis also showed continued higher PRRs among racial/ethnic minority groups compared to White respondents. Those 65+ reported lower rate ratios of barriers (PRR = 0.54; 95% CI: 0.50–0.57) compared to younger respondents, while women reported higher compared to men (PRR = 1.23; 95% CI: 1.18–1.28). Compared to those who were married, those who were divorced/separated, widowed, or never married reported higher rate ratios of barriers to care. By education status, those with less than a high school education reported the highest rate (PRR = 1.80; 95% CI: 1.69–1.92) compared to those with a college or technical school degree. Those who were unemployed (PRR = 1.60; 95% CI: 1.49–1.71), unable to work (PRR = 1.40; 95% CI: 1.32–1.48), or homemakers (PRR = 1.11; 95% CI: 1.03–1.21) reported the greatest barriers compared to those who were employed for wages, while those who were students or retired reported lower risk. Respondents in states that had not expanded Medicaid at the time of the survey had a higher PRR (1.22; 95% CI: 1.17–1.27)

## DISCUSSION

Our cross‐sectional study, using data from the 2018 BRFSS across eight states, found higher proportions of those living in nonmetropolitan areas, both noncore and micropolitan, lacking health insurance and experiencing medical debt, and changing their adherence to medications due to cost compared to their metropolitan counterparts. The majority of micropolitan respondents experienced at least one barrier to care, with non‐White respondents, particularly non‐Hispanic Black and Hispanic respondents, experiencing the greatest number of barriers. After accounting for other sociodemographic and state factors, we also found that those living in micropolitan areas tended to experience the most barriers. Our study also revealed that women, those with less education, those who are unemployed or not able to work, and racial/ethnic minorities experienced greater barriers.

Our findings on the disproportionate barriers facing those living in both micropolitan and noncore nonmetropolitan areas are consistent with prior evidence.^23^ Studies consistently have shown that more nonmetropolitan individuals lack health insurance compared to metropolitan individuals.^18^ Similarly, we found that nonmetropolitan populations were more likely to experience medical debt and were more likely to not take medications due to costs.^18^, ^24^ Additionally, our findings indicate that nonmetropolitan populations as well as racial/ethnic minorities in nonmetropolitan areas experience multiple access barriers at higher frequencies than their counterparts, underscoring the importance of systemic solutions to reduce the number of these barriers.

Even after controlling for other sociodemographic factors, we found that those living in micropolitan counties experienced greater barriers compared to those in metropolitan counties. Surprisingly, those living in more remote, noncore counties experienced no differences in the number of barriers than their metropolitan counterparts. Previous studies have shown that micropolitan local health departments receive less funding per capita than noncore departments, which may be reflective of a resource gap disproportionately affecting larger nonmetropolitan communities.^25^ However, previous studies tend to show either no differences between noncore and micropolitan counties or that noncore counties experienced greater barriers to care. These findings may reflect the unique distribution of noncore and micropolitan counties and experiences within the eight states who administered this optional survey.^20^, ^26^, ^27^ Future studies should continue to examine the nuances of nonmetropolitan differences in the number of barriers to care.

Our findings on the multiple barriers racial/ethnic minorities experience in access to care suggest the urgent needs to address medical debts, insurance coverage, and affordability for medication in ongoing efforts to improve health inequities.^28^ To address the accessibility, affordability, and acceptability of health care among nonmetropolitan residents simultaneously, a multifactorial and comprehensive approach is necessary with the aims of promoting health equity and mitigating the negative impacts of social determinants of health. In addition to the aforementioned Medicaid expansion and the 340B drug program, state‐level public health agencies and community‐based or faith‐based organizations can play a crucial role in promoting equitable access to quality, affordable health care, and other essential social services such as education, housing, transportation, and childcare.^29^ To ensure accessibility to these services, our findings highlight the need to prioritize nonmetropolitan and racial/ethnic minorities, as well as those who may lack financial and social capital.

We found that a higher number of barriers were present among those who lived in states that had to expand Medicaid. Medicaid expansion can be a critical way to improve access to care and reduce cost barriers, as expansion has been especially beneficial to nonmetropolitan residents.^30^, ^31^ Studies have shown that states that have expanded Medicaid have experienced reductions in medical debt, improvement in the percent of the population with health insurance, and less delayed care due to cost.^32^, ^33^ Beyond Medicaid expansion, other policy interventions such as maintaining the 340B program, which supports discount medications for nonmetropolitan hospitals and other settings, and regulating private and public insurance to reduce out‐of‐pocket costs are imperative to mitigate these barriers that disproportionately impact nonmetropolitan populations.^34^

### Limitations

The study had several limitations regarding the use of 2018 BRFSS data. First, the data relied on self‐reporting, which could lead to biases such as recall and response biases when respondents reported on access to care barriers. Second, the study only included noninstitutionalized adults randomly selected from landline and/or cell phone users, so the rural–urban differences in access to care barriers might not apply to other groups, such as children, homeless adults, or those in institutionalized settings including nursing homes, institutionalized medical facilities, incarceration facilities, homeless shelters, and so forth. Additionally, our results may not be generalizable to the entire US population, as only some states chose to participate, with some regions underrepresented. In particular, the data included only one state (New Hampshire) from the Northeast region and one state (Nebraska) from the Midwest region, whereas multiple states were included from the West and South regions. We hope that more states will participate in future BRFSS health care access surveys. The study also lacked data on some aspects of access to care. While our study examines the affordability, accommodation, and acceptability domains of 5 A's barriers to care, we were unable to measure travel burden (accessibility) and neighborhood‐level provider supply (availability) given the lack of granular geographic identifiers in the public use BRFSS data. Despite these limitations, the study used five access to care questions to compare the number of barriers experienced by metropolitan, nonmetropolitan micropolitan, and noncore respondents, providing essential information for addressing multiple barriers to accessing care.

## CONCLUSIONS

The findings from this study of BRFSS data from eight states are instructive for policymakers as they create programs and policies to reduce barriers to access and improve the affordability of health care. Adherence to medications, for example, or other medical interventions to manage chronic diseases is only accomplished if they are accessible and affordable. Medicaid expansion, the 340B drug program, as well as community‐based or faith‐based organizations can play a crucial role in promoting equitable access to quality, affordable health care, and other essential social services. This study provides timely information for multiple barriers to care across levels of rurality, highlighting the need to prioritize nonmetropolitan and racial/ethnic minorities, as well as those who may lack financial and social capital. Future research would benefit from more states having the funding to add this module to their BRFSS.

## CONFLICT OF INTEREST STATEMENT

Dr. Zahnd currently serves as the chair of the *Journal of Rural Health*’s editorial board. Drs. Crouch and Eberth are both also members of the *Journal of Rural Health* editorial board.

## Data Availability

The data that support the findings of this study are available in BRFSS at https://www.cdc.gov/brfss/annual_data/annual_2018.html. These data were derived from the following resources available in the public domain: BRFSS, https://www.cdc.gov/brfss/annual_data/annual_2018.html.
